# Body Mass Index underestimates excess adiposity: diagnostic discrepancy with bioelectrical impedance analysis and misclassification of nutritional status

**DOI:** 10.3389/fendo.2026.1845457

**Published:** 2026-06-22

**Authors:** Rodrigo Yáñez-Sepúlveda, Carlos Abraham Herrera-Amante, César Octavio Ramos-García, Mario Muñoz-López, Vicente Javier Clemente-Suárez, José Francisco Tornero-Aguilera

**Affiliations:** 1 Facultad de Educación y Humanidades. Escuela de Ciencias del Deporte, Universidad Andres Bello, Viña del Mar, Chile; 2 School of Medicine, Universidad Espíritu Santo, Samborondón, Ecuador; 3 Nutritional Assessment and Nutritional Care Laboratory, Division of Health Sciences, Tonalá University Center, University of Guadalajara, Tonalá, Mexico; 4 Ibero-American Network of Researchers in Applied Anthropometry, Almería, Spain; 5 Department of Sport Sciences, Faculty of Sport and Health Sciences, Fit Generation Research Institute, Andorra la Vella, Andorra; 6 Grupo de Investigación en Cultura, Educación y Sociedad, Universidad de la Costa, Barranquilla, Colombia

**Keywords:** machine learning, obesity abdominal, body fat distribution, body mass index, obesity

## Abstract

**Background:**

Body mass index (BMI) remains the most widely used tool for obesity screening in clinical and public health settings due to its simplicity and accessibility. However, as BMI does not directly quantify adiposity, it may fail to detect excess body fat and obscure clinically relevant metabolic phenotypes. This limitation has not been adequately characterized in large Latin American cohorts. We quantified diagnostic discordance between BMI- and bioelectrical impedance analysis (BIA)-based obesity classification in Chilean and Mexican adults using age- and sex-specific body fat percentage thresholds.

**Methods:**

In this cross-sectional study, we pooled data from three adult body-composition databases, including 12,604 participants from Chile and Mexico. Adiposity-defined obesity was classified using age- and sex-specific body fat percentage cut-offs proposed by Gallagher et al., whereas BMI-defined obesity followed World Health Organization criteria (BMI ≥30 kg/m²). Participants were allocated to four phenotypes: concordant non-obese, normal-weight obesity, high-BMI/normal-adiposity, and concordant obese. Agreement between BMI and BIA classifications was evaluated using Cohen’s kappa, sensitivity, specificity, and Bland-Altman analysis. Ten supervised machine learning algorithms were trained and internally validated by 10-fold cross-validation to predict BIA-defined obesity using anthropometric variables.

**Results:**

Agreement between BMI and BIA was moderate (κ = 0.443). BMI showed high specificity (96.9%) but low sensitivity (46.3%), failing to identify 53.7% of BIA-defined obesity. Misclassification was more pronounced in women (κ = 0.386). Normal-weight obesity was identified in 16.2% of women and 4.6% of men. Among men with elevated BMI (≥ 25 kg/m²), 42.8% showed the high-BMI/normal-adiposity phenotype. Ordinary least squares models explained 68.6%–70.6% of body fat variance, with systematic error at extremes. Machine learning models showed high discrimination, with multilayer perceptron achieving AUC = 0.999.

**Conclusion:**

BMI underestimates excess adiposity in this Latin American adult sample and misclassifies relevant body composition phenotypes, particularly in women. Reliance on BMI alone may obscure a large proportion of individuals with excess adiposity, potentially limiting early detection and preventive strategies. Predictive models using simple anthropometric inputs may improve adiposity screening where direct body composition assessment is unavailable.

## 1 Introduction

Body mass index (BMI), originally derived from the Quetelet index, remains the most widely used screening tool for overweight and obesity in both clinical practice and population surveillance because it is simple, inexpensive, and based only on body weight and height (1). The World Health Organization defines adult overweight as a BMI ≥ 25 kg/m² and obesity as a BMI ≥ 30 kg/m² (2). However, BMI is an indirect proxy for adiposity rather than a direct measure of body composition and cannot distinguish fat mass from lean soft tissue, bone mass, or body water (3). Consequently, individuals with identical BMI values may differ substantially in body fat percentage, fat distribution, and cardiometabolic risk (4).

These limitations have driven interest in body composition assessment methods that provide a more physiologically meaningful estimate of adiposity. Bioelectrical impedance analysis (BIA) is a widely used, noninvasive technique for estimating body composition, including fat-free mass, total body water, and derived fat mass when applied under standardized conditions and appropriate prediction models (5). Although BIA is not a criterion method equivalent to multicomponent models or dual-energy X-ray absorptiometry, it provides greater biological specificity than BMI and is considerably more feasible for large-scale and clinical applications (6). In this context, Gallagher et al. proposed age-, sex-, and ethnicity-adjusted body fat percentage thresholds linked to BMI categories, providing a framework to interpret adiposity beyond weight-for-height indices alone (7).

The discordance between BMI-defined obesity and adiposity-defined obesity has important clinical implications, as it generates phenotypes that remain undetected by conventional screening approaches (8). One such phenotype is normal-weight obesity, referring to individuals with BMI within the normal range but elevated body fat percentage, a condition associated with adverse cardiometabolic profiles despite apparently normal body weight (9, 10). Conversely, BMI may classify individuals as overweight or obese despite relatively low adiposity, particularly in those with higher lean mass, leading to potential overestimation of obesity in specific subgroups (11). Collectively, these discrepancies highlight fundamental limitations of BMI as a surrogate marker of adiposity at the individual level.

These issues are particularly relevant in Latin America, where obesity and related noncommunicable diseases have increased substantially in recent decades (12). The region is undergoing rapid nutritional and epidemiological transitions characterized by dietary westernization, reduced physical activity, and population aging, all of which contribute to rising adiposity and cardiometabolic burden (13). Despite this context, BMI remains the primary tool for clinical screening, epidemiological surveillance, and public health policy across the region, largely due to its simplicity and low cost (14). This reliance may obscure a substantial proportion of individuals with excess adiposity who are not identified using standard BMI thresholds alone.

In parallel, the integration of machine learning approaches into clinical and epidemiological research offers an opportunity to improve adiposity classification using routinely collected data (15). If sufficient predictive signal exists within basic anthropometric variables, algorithmic models may approximate adiposity-defined obesity without the need for direct body composition assessment. This approach is particularly relevant in settings where access to BIA or other body composition techniques is limited and where scalable screening strategies are required.

Accordingly, the present study evaluated the diagnostic concordance between BMI and BIA-based adiposity classification in a large cohort of Chilean and Mexican adults using World Health Organization BMI criteria and the age- and sex-specific body fat percentage thresholds proposed by Gallagher et al. We further quantified the prevalence of discordant phenotypes, including normal-weight obesity and BMI-defined obesity without excess adiposity, and assessed the extent to which BMI misclassifies adiposity at the individual level. Finally, we examined whether supervised machine learning models based on simple anthropometric variables could accurately predict BIA-defined obesity in the absence of direct body composition measurements.

## 2 Materials and methods

### 2.1 Study design and data sources

This cross-sectional study integrated data from three independent bioelectrical impedance analysis (BIA) datasets collected between 2018 and 2023. The analytical cohort included civilian adults from Quintero (Valparaíso Region, Chile) and Guadalajara (Jalisco, Mexico).

Body composition measurements were obtained using InBody^®^ 370 and 720 analyzers (Biospace Co., Seoul, South Korea), which are multifrequency, segmental BIA devices widely used in clinical and research settings (16). Methodological aspects specific to multifrequency segmental BIA (multiple-frequency current injection at body-segment level, with separate impedance estimation for trunk and limbs) are reviewed in detail elsewhere (17). All measurements were conducted following standardized pre-assessment conditions to reduce variability associated with hydration status and recent physical activity. Participants were instructed to fast for at least two hours, void their bladder prior to measurement, wear light clothing, and avoid strenuous physical activity for at least 12 hours before assessment (18).

The final analytical sample consisted of 12,604 participants, after excluding individuals under 18 years of age, those over 80 years of age, and those with missing data on key body composition variables. The pooled analytical cohort was derived from three independent body-composition databases. The Chilean sub-cohort (n = 7,349 adults) was obtained from two datasets collected in Quintero (Valparaíso Region) between 2018 and 2023 using an InBody^®^ 370 analyzer. The Mexican sub-cohort (n = 5,255 adults) was obtained from a single dataset collected in Guadalajara (Jalisco) using an InBody^®^ 720 analyzer. All three datasets applied identical pre-measurement standardization protocols (fasting, voided bladder, light clothing, no strenuous activity in the preceding 12 hours), and harmonization across datasets was performed at the variable level, retaining only the common anthropometric and body-composition fields produced by both InBody models. The InBody 370 and 720 share the same eight-point tetrapolar electrode configuration and multifrequency segmental measurement principle, and previous cross-device comparisons have reported close agreement in body-composition outputs between the two instruments (16). Because both devices were used in geographically distinct sub-cohorts, residual device-related variability cannot be ruled out, and country was retained as a covariate in stratified analyses to account for joint device-population effects.

### 2.2 Obesity classification criteria

Adiposity status was assessed using both BMI-based and body fat percentage–based criteria.

Body mass index (BMI) was calculated as body weight in kilograms divided by height in meters squared (kg/m²). Participants were classified according to World Health Organization criteria as underweight (< 18.5 kg/m²), normal weight (18.5–24.9 kg/m²), overweight (25.0–29.9 kg/m²), or obese (≥ 30.0 kg/m²) (2).

Adiposity-defined obesity was determined using age- and sex-specific body fat percentage (BF%) thresholds proposed by Gallagher et al. (7). These thresholds account for known differences in fat distribution across age and sex and were derived from a multiethnic adult sample. The applied cut-offs were as follows:

- Men: ≥ 25% (20–39 years), ≥ 28% (40–59 years), ≥ 30% (60–79 years).
- Women: ≥ 35% (20–39 years), ≥ 38% (40–59 years), ≥ 42% (60–79 years).

A complete summary of classification thresholds is presented in **Table 1**.

**Table 1:** Classification cut-off values: WHO BMI and Gallagher BF% criteria.

System	Category	Men	Women	Age group	Reference
WHO BMI	Underweight	< 18.5 kg/m²	< 18.5 kg/m²	All	(2)
WHO BMI	Normal weight	18.5–24.9 kg/m²	18.5–24.9 kg/m²	All	(2)
WHO BMI	Overweight	25.0–29.9 kg/m²	25.0–29.9 kg/m²	All	(2)
WHO BMI	Obese	≥ 30.0 kg/m²	≥ 30.0 kg/m²	All	(2)
Gallagher BF%	Obese	≥ 25%	≥ 35%	20–39 years	(7)
Gallagher BF%	Obese	≥ 28%	≥ 38%	40–59 years	(7)
Gallagher BF%	Obese	≥ 30%	≥ 42%	60–79 years	(7)

BF%, body fat percentage; BMI, body mass index.

### 2.3 Phenotype classification

Participants were classified into four mutually exclusive phenotypic categories based on the combined application of BMI and BF% criteria:

- Concordant non-obese: BMI < 25 kg/m² and BF% below Gallagher threshold.
- Normal-weight obesity (NWO): BMI < 25 kg/m² and BF% ≥ Gallagher threshold.
- High-BMI/normal-adiposity phenotype: BMI ≥ 25 kg/m² and BF% below Gallagher threshold.
- Concordant obese: BMI ≥ 25 kg/m² and BF% ≥ Gallagher threshold.

This classification framework was used to quantify the extent of discordance between BMI-based and adiposity-based definitions of obesity. The BMI threshold of 25 kg/m² was deliberately adopted for phenotype classification rather than the obesity threshold of 30 kg/m² to ensure that the discordant phenotypes captured the full spectrum of weight-for-height misclassification, including individuals in the overweight range whose adiposity status diverges from BMI-based labelling. By contrast, the diagnostic concordance analysis in Section 2.4.2 retained the conventional World Health Organization obesity threshold (BMI ≥ 30 kg/m²) so that the operating characteristics of BMI as an obesity screening tool could be evaluated against the reference standard exactly as it is applied in clinical and public-health practice. The two thresholds therefore serve distinct analytical purposes (phenotypic structure versus diagnostic test performance) and are not interchangeable.

### 2.4 Statistical analysis

#### 2.4.1 Descriptive statistics and normality assessment

Continuous variables are presented as mean ± standard deviation (SD), and categorical variables as counts and proportions. Normality of continuous variables was assessed using the Shapiro–Wilk test (19). Given the sensitivity of normality tests in large samples, additional assessments were conducted using random subsampling (n = 500) and visual inspection of distributions. When non-normality was detected, non-parametric methods were used for group comparisons.

#### 2.4.2 Diagnostic agreement

Agreement between BMI-based obesity (BMI ≥ 30 kg/m²) and adiposity-defined obesity (BF% thresholds) was quantified using Cohen’s kappa coefficient (20). Strength of agreement was interpreted according to established benchmarks: < 0.20 slight, 0.21–0.40 fair, 0.41–0.60 moderate, 0.61–0.80 substantial, and 0.81–1.00 near-perfect (21). Sensitivity, specificity, positive predictive value (PPV), and negative predictive value (NPV) were calculated considering BF%-based classification as the reference standard. To evaluate agreement at the continuous level, Bland–Altman analysis was performed using standardized z-scores of BMI and BF%, allowing assessment of proportional bias and limits of agreement (mean difference ± 1.96 SD) (22).

#### 2.4.3 Regression analysis

Ordinary least squares (OLS) regression models were fitted to evaluate the relationship between BMI and BF% (BF% as dependent variable, BMI as predictor). Models were stratified by sex and country. Model performance was assessed using the coefficient of determination (R²).

Residual diagnostics were performed to evaluate model assumptions, including heteroscedasticity and distributional properties. Departures from normality and homoscedasticity were interpreted as evidence of non-linear or non-uniform relationships between BMI and adiposity across the distribution rather than as purely statistical artifacts.

#### 2.4.4 Machine learning models

Supervised machine learning models were developed to predict adiposity-defined obesity (binary outcome based on BF% thresholds). Predictor variables included age, weight (kg), height (cm), BMI (kg/m²), basal metabolic rate (kcal/day), sex, and country.

All models were implemented using scikit-learn (version consistent with current stable release) within a standardized pipeline including z-score normalization via StandardScaler (23). Model performance was evaluated using stratified 10-fold cross-validation to preserve class distribution across folds and reduce overfitting bias.

Evaluation metrics included area under the receiver operating characteristic curve (AUC-ROC), average precision, F1-score, precision, recall, specificity, Brier score, Cohen’s kappa, and Matthews correlation coefficient.

To address the predictor redundancy inherent to the full feature set, two pre-specified sensitivity analyses were conducted. In the first, BMI was removed from the predictor matrix, retaining age, body weight, height, basal metabolic rate, sex, and country, in order to quantify the discriminatory performance attributable to information not mathematically encoded by the BMI value. In the second, both BMI and BMR were removed, leaving only age, body weight, height, sex, and country, to evaluate the minimum predictor set sufficient to approximate adiposity-defined obesity. All models were retrained and re-evaluated under the same 10-fold stratified cross-validation pipeline, and discrimination metrics were compared with the full-feature reference. Because direct external validation in an independent cohort was not feasible with the present data, internal validation was complemented by stratified analyses across sex and country sub-cohorts to assess the stability of model performance across population strata.

#### 2.4.5 Feature importance and model interpretability

Feature importance was assessed using three complementary approaches:

1. Permutation-based importance calculated as the decrease in model performance (ΔAUC) after random permutation of each feature.
2. Mean decrease in impurity (Gini importance) derived from the Random Forest model.
3. Repeated permutation importance (n = 20 iterations) to assess stability of estimates.

Consistency across methods was interpreted as evidence of robust predictor relevance.

Partial dependence plots were generated for the most influential predictors to visualize non-linear relationships between individual variables and predicted probability of adiposity-defined obesity.

### 2.5 Ethical considerations

The data from Chile included in the study are part of a longitudinal project conducted at the Quintero Physical Fitness Assessment Laboratory, led by Dr. Rodrigo Yáñez Sepúlveda. The data from Mexico come from a longitudinal study led by Drs. Herrera and Ramos at the LECEN of the University of Guadalajara. Both studies have taken into account the ethical and legal considerations of each country, and these studies are part of the multicenter collaboration of the Ibero-American Network of Researchers in Applied Anthropometry https://riba2.org/.

## 3 Results

### 3.1 Cohort characteristics and phenotype distribution

The final analytical cohort included 12,604 Latin American adults, with a balanced sex distribution (6,328 women, 50.2%) and a mean age of 30.3 ± 11.6 years.

Marked differences were observed between BMI-based and adiposity-based classifications of obesity. Using the age- and sex-specific body fat percentage thresholds proposed by Gallagher et al., the prevalence of adiposity-defined obesity was 47.2%. In contrast, application of World Health Organization BMI criteria (BMI ≥ 30 kg/m²) identified 23.5% of participants as obese, corresponding to an absolute difference of 23.7 percentage points.

**Figure 1:**
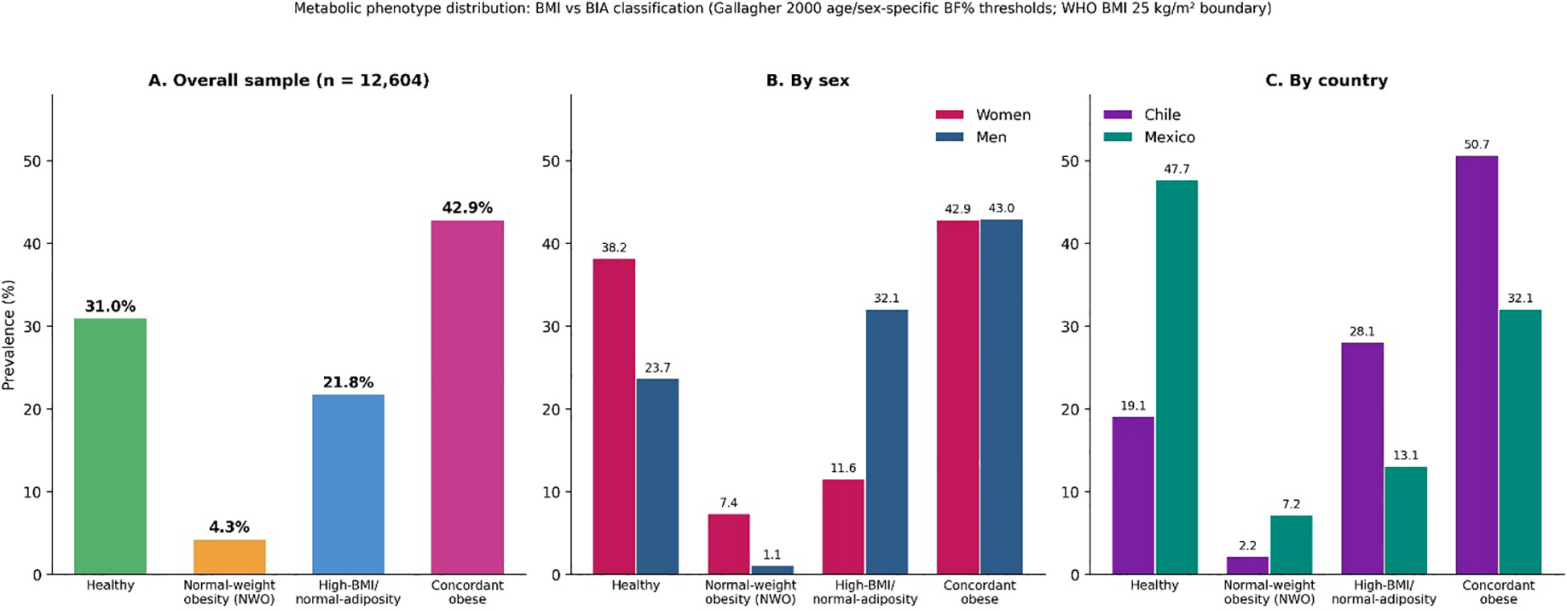
Distribution of BMI–BF% phenotypes in the overall sample and stratified by sex and country. **(A)** Overall cohort (n = 12,604). **(B)** Distribution by sex. **(C)** Distribution by country. Phenotypes were defined using World Health Organization BMI thresholds and age- and sex-specific body fat percentage cut-offs proposed by Gallagher et al. The concordant non-obese phenotype is labeled ‘healthy’ in the figure panels. Normal-weight obesity (NWO) was more prevalent in women, whereas the high-BMI/normal-adiposity phenotype was more prevalent in men. A higher prevalence of adiposity-defined obesity was observed in the Chilean sub-cohort compared with the Mexican sub-cohort.

When participants were categorized according to combined BMI and BF% criteria, substantial heterogeneity in phenotype distribution was observed (**Figure 1**, **Table 2**). The concordant obese group (BMI ≥ 25 kg/m² and BF% ≥ threshold) represented 42.9% of the cohort.

**Table 2:** Descriptive statistics by phenotype and sex (mean ± SD).

Phenotype/Sex	n	Age (yr)	BMI (kg/m²)	BF%	Weight (kg)	Height (cm)	SMM (kg)	BMR (kcal)
Concordant non-obese — Women	2,415	24.2 ± 8.2	21.5 ± 2.1	27.7 ± 4.9	55.9 ± 6.9	161.0 ± 6.0	21.9 ± 3.0	1,240 ± 106
Concordant non-obese — Men	1,490	27.0 ± 8.3	22.8 ± 1.8	16.4 ± 4.3	70.9 ± 8.3	176.3 ± 7.0	33.5 ± 4.0	1,648 ± 146
NWO — Women	466	24.6 ± 6.7	23.6 ± 1.2	37.3 ± 2.0	59.8 ± 5.1	158.9 ± 5.1	20.2 ± 2.0	1,180 ± 73
NWO — Men	72	29.1 ± 10.7	24.1 ± 1.0	27.6 ± 1.7	70.2 ± 5.0	170.4 ± 4.3	28.4 ± 2.3	1,468 ± 84
High-BMI/normal-adiposity phenotype — Women	731	33.8 ± 12.9	26.9 ± 1.7	32.6 ± 3.6	70.1 ± 7.0	161.3 ± 5.5	26.2 ± 3.2	1,391 ± 114
High-BMI/normal-adiposity phenotype — Men	2,017	36.2 ± 11.3	27.4 ± 1.7	22.0 ± 3.7	83.5 ± 8.2	174.5 ± 6.2	37.2 ± 3.9	1,775 ± 142
Concordant obese — Women	2,716	31.5 ± 12.1	31.0 ± 5.0	42.8 ± 4.9	78.9 ± 13.5	159.6 ± 6.0	24.6 ± 3.3	1,336 ± 119
Concordant obese — Men	2,697	34.5 ± 11.0	30.8 ± 3.5	32.2 ± 4.8	92.6 ± 12.4	173.3 ± 6.3	35.5 ± 4.4	1,719 ± 157

NWO, normal-weight obesity; BF%, body fat percentage; SMM, skeletal muscle mass; BMR, basal metabolic rate. All continuous variables are expressed as mean ± standard deviation.

Discordant phenotypes were also frequent and showed clear sex-specific patterns (**Figure 1**). The high-BMI/normal-adiposity phenotype (BMI ≥ 25 kg/m² with BF% below threshold) accounted for 21.8% of the total sample and was more prevalent in men than in women (2,017 men vs. 731 women; ratio 2.8:1). In contrast, the normal-weight obesity (NWO) phenotype (BMI < 25 kg/m² with BF% ≥ threshold) represented 4.3% of the cohort overall and was predominantly observed in women, with an approximate female-to-male ratio of 6.5:1.

Descriptive statistics by phenotype and sex are presented in **Table 2**. Across phenotypic groups, individuals classified as concordant obese exhibited the highest body fat percentage and body weight, whereas the high-BMI/normal-adiposity phenotype was characterized by elevated BMI values accompanied by comparatively lower body fat percentages and higher skeletal muscle mass, particularly in men.

Geographical differences were also identified (**Figure 1**). The prevalence of adiposity-defined obesity was higher in the Chilean sub-cohort (52.9%) compared with the Mexican sub-cohort (39.3%).

### 3.2 Distribution of body fat percentage across BMI categories

**Figure 2:**
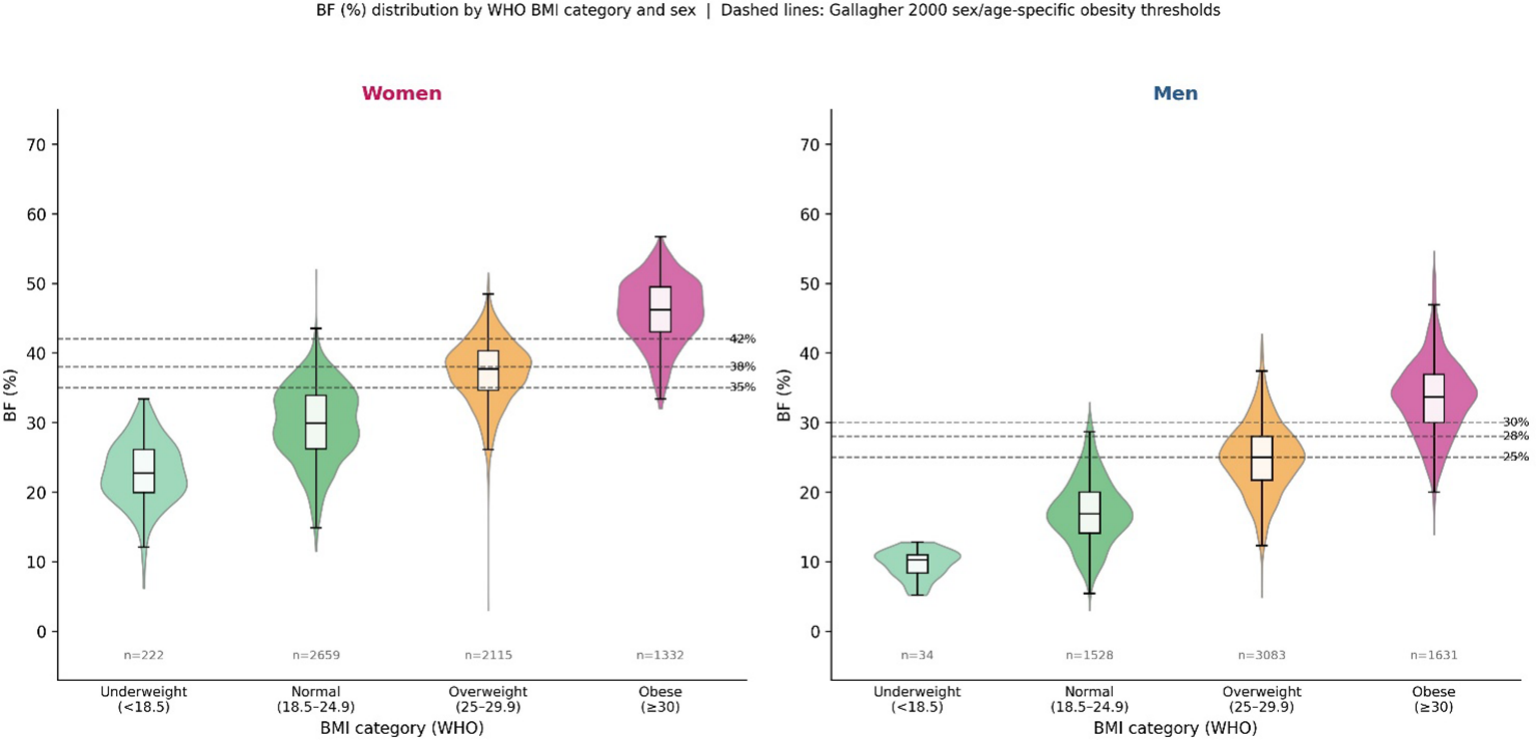
Distribution of BF% across BMI categories stratified by sex. Violin and box plots illustrate BF% within each BMI category (underweight, normal weight, overweight, and obese) for women and men. Dashed horizontal lines represent Gallagher age-adjusted obesity thresholds. Overlap in BF% distributions across adjacent BMI categories is observed, particularly among women.

Body fat percentage distributions across World Health Organization BMI categories, stratified by sex, are shown in **Figure 2**. Substantial overlap in BF% values was observed between adjacent BMI categories in both sexes, indicating heterogeneity in adiposity within each BMI-defined group.

In women, individuals classified as having normal BMI exhibited BF% values extending into the obesity range according to Gallagher thresholds. The mean BF% within the BMI-normal category (18.5–24.9 kg/m²) was 29.8 ± 5.5%, compared with the threshold of 35%. In men, the corresponding value was 17.1 ± 4.7% versus a threshold of 25%.

Across BMI categories, BF% increased progressively from underweight to obese groups; however, dispersion within each category remained considerable. Notably, BF% values in the overweight and obese BMI categories overlapped with adjacent strata, particularly in women, suggesting that BMI categories do not fully capture variability in adiposity at the individual level.

### 3.3 BMI–BF% relationship and phenotype quadrant distribution

**Figure 3:**
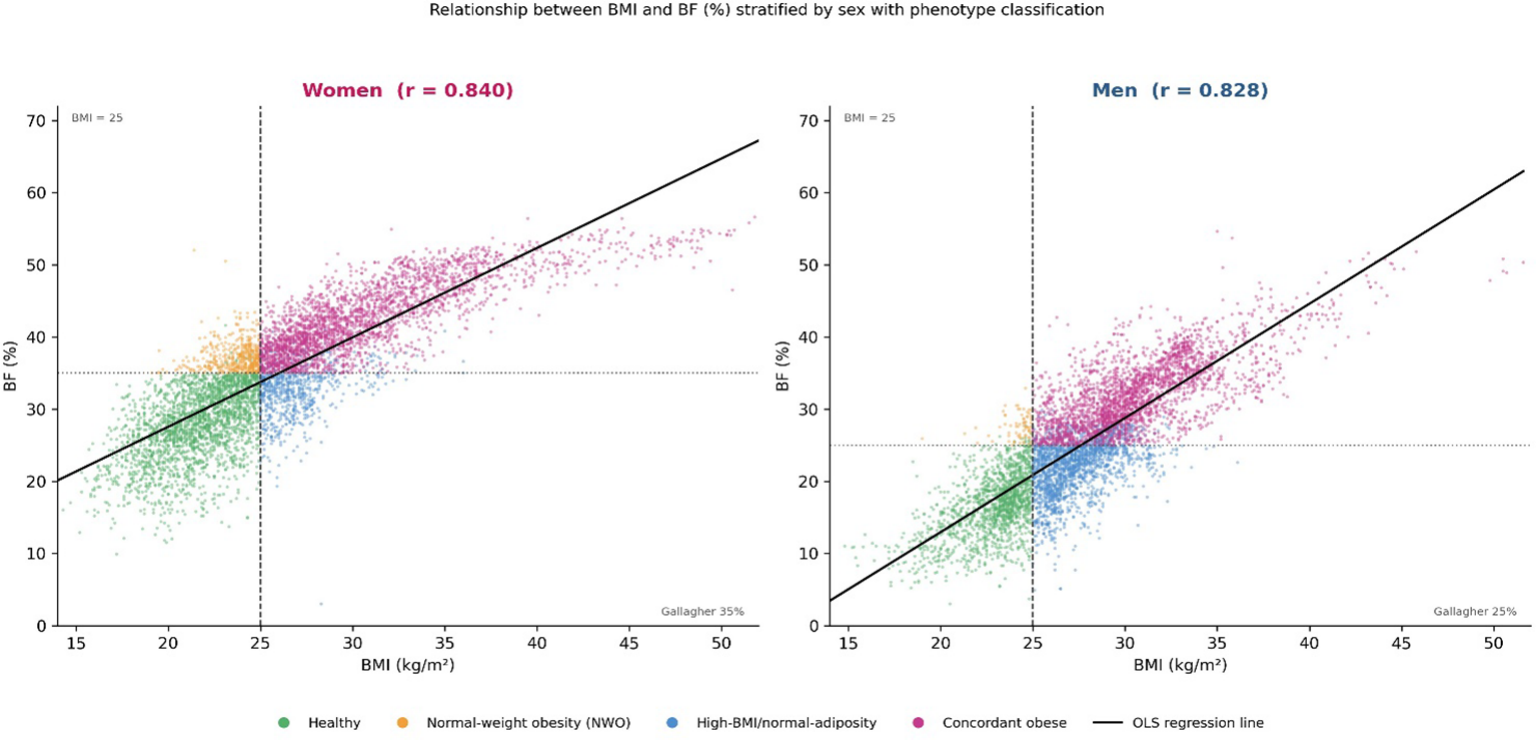
Relationship between BMI and BF% stratified by sex with phenotype classification. Scatter plots display BMI (x-axis) and BF% (y-axis) for women and men. Vertical dashed lines indicate the BMI threshold of 25 kg/m², and horizontal dotted lines represent sex-specific BF% thresholds based on Gallagher criteria. Points are colored according to phenotype classification, with the concordant non-obese phenotype labeled ‘healthy’ in the legend. Solid lines represent ordinary least squares regression fits. Distinct clusters corresponding to discordant phenotypes are observed in the upper-left (normal-weight obesity) and lower-right (high-BMI/normal-adiposity) quadrants.

The relationship between BMI and BF%, stratified by sex, is shown in **Figure 3**. A strong positive association between BMI and BF% was observed in both women (r = 0.840, p < 0.001) and men (r = 0.828, p < 0.001), as indicated by the ordinary least squares regression lines.

Despite this overall association, substantial dispersion was evident around the regression line, particularly near clinically relevant threshold regions. The quadrant-based classification revealed distinct clusters of individuals with discordant BMI and adiposity classifications.

In the upper-left quadrant (BMI < 25 kg/m² with BF% above threshold), corresponding to the normal-weight obesity (NWO) phenotype, a clearly defined cluster of individuals was observed, particularly in women. Conversely, in the lower-right quadrant (BMI ≥ 25 kg/m² with BF% below threshold), a second cluster representing the high-BMI/normal-adiposity phenotype was identified, predominantly in men.

These discordant regions indicate that, although BMI and BF% are strongly correlated at the population level, classification based on BMI alone does not consistently reflect individual adiposity status.

### 3.4 BMI–BF% regression and residual diagnostics

Ordinary least squares regression models examining the relationship between BMI and BF% are summarized in **Table 3**. Across all models, BMI explained a substantial proportion of the variance in BF%, with coefficients of determination of 0.706 in women and 0.686 in men; within-sex models stratified by country showed comparable fit (R² 0.674–0.691).

**Table 3:** Ordinary least squares regression models of body fat percentage on BMI, stratified by sex and by sex within country.

Model	β_0_	β_1_ (BMI)	R²	SE	F-stat	p-value	Shapiro-Wilk p
BF% ~ BMI (Women)	2.772	1.239	0.706	0.010	15,219	< 0.001	< 0.001
BF% ~ BMI (Men)	-18.697	1.582	0.686	0.014	13,708	< 0.001	< 0.001
BF% ~ BMI (Women, Chile)	5.775	1.130	0.691	0.014	6,459	< 0.001	< 0.001
BF% ~ BMI (Men, Chile)	-19.366	1.605	0.674	0.017	9,220	< 0.001	< 0.001
BF% ~ BMI (Women, Mexico)	-0.578	1.380	0.679	0.016	7,288	< 0.001	< 0.001
BF% ~ BMI (Men, Mexico)	-17.761	1.549	0.681	0.025	3,866	< 0.001	0.005

β_0_, intercept; β_1_, BMI coefficient; R², coefficient of determination; SE, standard error of β_1_.

Sex-stratified models revealed differences in both intercepts and slopes. In women, the estimated increase in BF% per unit increase in BMI was 1.239 (intercept: 2.772), whereas in men it was 1.582 (intercept: −18.697). These differences indicate that the relationship between BMI and BF% is sex-dependent.

**Figure 4:**
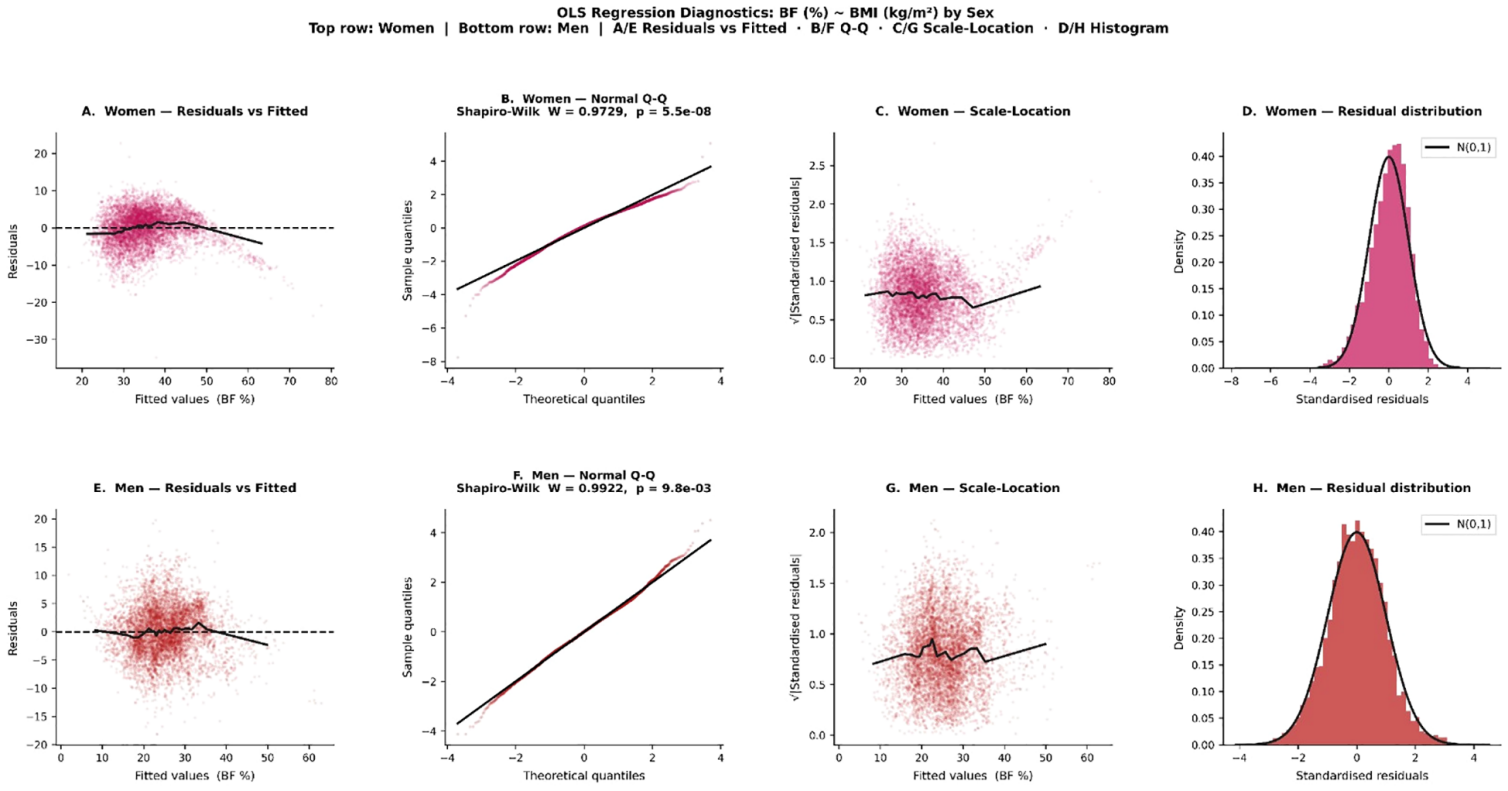
Residual diagnostics for OLS models of BF% as a function of BMI stratified by sex. Diagnostic plots for women include **(A)** residuals versus fitted values, **(B)** normal Q-Q plots, **(C)** scale-location plots, and **(D)** residual histograms. Corresponding plots for men are shown in **(E)** residuals versus fitted values, **(F)** normal Q-Q plots, **(G)** scale-location plots, and **(H)** residual histograms. Deviations from normality and homoscedasticity are observed in both sexes, particularly at higher fitted values.

Residual diagnostics are presented in **Figure 4**. In both sexes, deviations from ideal model assumptions were observed.

Residuals versus fitted plots indicated non-uniform variance across the range of fitted values, particularly at higher BMI levels. Quantile–quantile plots showed departures from the theoretical normal distribution, with deviations at both lower and upper tails. Scale–location plots suggested heteroscedasticity, with increasing dispersion of residuals across fitted values. Residual histograms further indicated asymmetry relative to a normal distribution.

These findings were supported by Shapiro–Wilk tests, which indicated non-normality of residuals in all models (p < 0.01). Overall, the diagnostic analyses suggest that the relationship between BMI and BF% is not fully captured by a simple linear model, particularly at the extremes of body composition.

### 3.5 Bland–Altman agreement analysis

**Figure 5:**
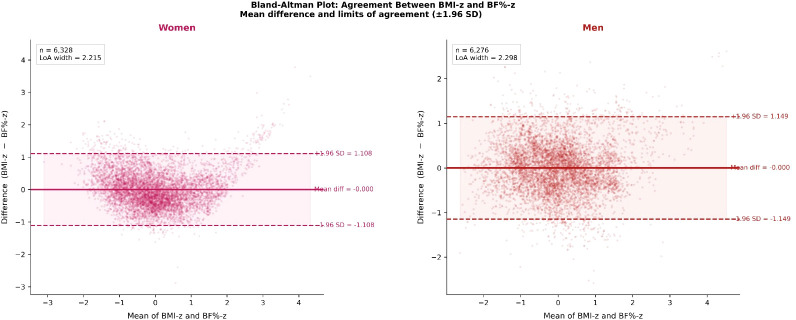
Bland–Altman plots showing agreement between standardized BMI and BF% values stratified by sex. The difference between BMI-z and BF%-z scores is plotted against their mean. The solid horizontal line represents the mean difference, and dashed lines indicate limits of agreement (± 1.96 SD). Wide limits of agreement and non-uniform patterns across the distribution indicate variability in agreement between BMI and BF%.

Agreement between standardized BMI and BF% values was evaluated using Bland–Altman analysis (**Figure 5**). By construction, the mean difference between BMI-z and BF%-z scores was approximately zero in both sexes. Because BMI and BF% measure related but conceptually distinct constructs, the conventional Bland–Altman framework cannot be applied to their native units, and the z-score transformation forces the mean difference toward zero by construction. The analysis is therefore presented as an exploratory descriptor of the structure of disagreement across the body-composition spectrum rather than as a formal method-comparison test, and should be interpreted alongside the kappa, sensitivity, specificity, and quadrant analyses presented in Sections 3.3 and 3.6, which directly evaluate diagnostic agreement.

However, the limits of agreement were wide (± 1.108 in women and ±1.149 in men), indicating substantial variability between BMI-based and adiposity-based measurements at the individual level.

Inspection of the plots revealed patterns consistent with proportional bias. At higher mean values, BMI tended to yield higher standardized values relative to BF%, whereas at lower mean values the opposite pattern was observed. This indicates that the discrepancy between BMI and BF% is not constant across the range of body composition.

These findings suggest that agreement between BMI and BF% varies across the distribution, with greater divergence observed at the extremes of body composition.

### 3.6 Diagnostic agreement between BMI-defined and adiposity-defined obesity

Agreement between BMI-defined obesity (BMI ≥ 30 kg/m²) and adiposity-defined obesity was evaluated using Cohen’s kappa coefficient (**Table 4**). Overall agreement was moderate (κ = 0.443).

**Table 4:** Diagnostic agreement between BMI-defined and adiposity-defined obesity classification.

Stratum	n	Cohen’s κ	Sensitivity	Specificity	PPV	NPV	Accuracy
Overall (all)	12,604	0.443	0.463	0.969	0.929	0.668	0.730
Women	6,328	0.386	0.403	0.984	0.962	0.620	0.692
Men	6,276	0.508	0.532	0.955	0.903	0.721	0.768
Chile	7,349	0.464	0.526	0.951	0.923	0.641	0.726
Mexico	5,255	0.374	0.344	0.988	0.948	0.700	0.735
Women Chile	2,884	0.393	0.495	0.956	0.948	0.539	0.671
Men Chile	4,465	0.511	0.552	0.948	0.905	0.704	0.761
Women Mexico	3,444	0.320	0.285	0.999	0.995	0.671	0.709
Men Mexico	1,811	0.484	0.468	0.968	0.894	0.758	0.784

Reference standard: adiposity-defined obesity based on age- and sex-specific BF% thresholds (Gallagher et al.).

BMI criterion for obesity: ≥ 30 kg/m² (World Health Organization).

Abbreviations: PPV, positive predictive value; NPV, negative predictive value; κ, Cohen’s kappa.

Approximate 95% confidence intervals for Cohen’s κ (bootstrap, 400 resamples): Overall 0.429–0.456; Women 0.370–0.403; Men 0.489–0.528; Chile 0.446–0.481; Mexico 0.352–0.396; Women Chile 0.368–0.421; Men Chile 0.485–0.535; Women Mexico 0.295–0.346; Men Mexico 0.439–0.522.

BMI showed low sensitivity for detecting adiposity-defined obesity (46.3%), indicating that a substantial proportion of individuals classified as obese by BF% were not identified using BMI criteria. In contrast, specificity was high (96.9%), indicating that BMI-defined obesity corresponded closely with adiposity-defined obesity when present.

Sex-stratified analyses showed lower agreement in women (κ = 0.386) than in men (κ = 0.508). Sensitivity was lower in women (40.3%) than in men (53.2%), whereas specificity remained high in both groups (≥ 95%).

Country-stratified analyses revealed a divergent pattern. Agreement was moderate in Chile (κ = 0.464) and fair in Mexico (κ = 0.374), with sensitivity of 52.6% and 34.4%, respectively, and specificity consistently high (≥ 95%).

Across all subgroups, sensitivity ranged from 28.5% to 55.2%, while specificity remained above 94%. These results indicate that BMI-based classification has limited sensitivity for detecting adiposity-defined obesity, despite high specificity. Because the Gallagher BF% thresholds vary across three age bands and the cohort skewed toward younger adults (mean age 30.3 ± 11.6 years), an exploratory age-stratified concordance analysis was performed, restricted to the 11,179 participants falling within the Gallagher 20–79-year bands (1,425 individuals aged 18–19 or ≥ 80 years fell outside these bands). The 20–39-year band, which represented the largest stratum (n = 8,248), showed metrics closely aligned with the overall estimates (κ = 0.380; sensitivity = 39.4%; specificity = 97.7%), confirming that the overall agreement pattern is driven primarily by this group. The 40–59-year band (n = 2,722) showed substantially higher agreement (κ = 0.619; sensitivity = 69.6%; specificity = 92.5%), and the 60–79-year band (n = 209) showed the highest agreement (κ = 0.762), reflecting the higher overlap between BMI and adiposity-based classifications at older ages. These results indicate that the diagnostic underperformance of BMI documented in the overall analysis is most pronounced in young adults, the age stratum in which screening for early metabolic risk is arguably most consequential.

### 3.7 Normal-weight obesity prevalence

Among individuals classified as having normal BMI (BMI < 25 kg/m²), the prevalence of normal-weight obesity (NWO) was 16.2% in women (466/2,881) and 4.6% in men (72/1,562).

**Figure 6:**
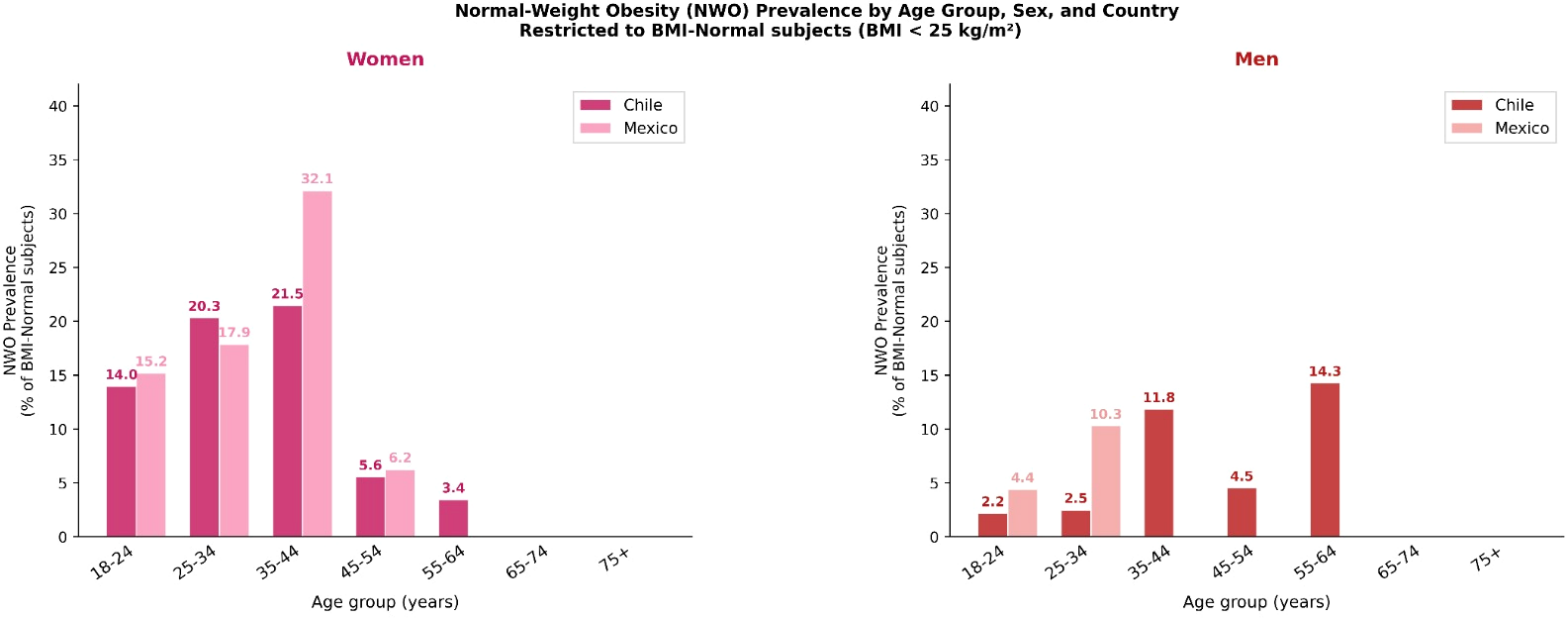
Prevalence of normal-weight obesity (NWO) by age group, sex, and country among individuals with BMI < 25 kg/m². Bar plots show NWO prevalence across age categories in women and men, stratified by country. NWO prevalence is higher in women than in men across all age groups, with peak values observed in mid-adulthood.

Age- and country-specific distributions are presented in **Figure 6**. In women, NWO prevalence was highest in the 35–44 age group and remained elevated across early and mid-adulthood. Differences between countries were observed, with variation in NWO prevalence across age groups, although no consistent pattern was evident across all strata.

In men, NWO prevalence remained low across all age groups, generally below 6%, with modest variation between age categories.

Overall, NWO was substantially more prevalent in women than in men across all age groups.

### 3.8 Machine learning performance

Performance metrics for all models are summarized in **Table 5**. The multilayer perceptron (MLP) achieved the highest performance, with an AUC of 0.999, F1-score of 0.983, and Cohen’s κ of 0.967. Logistic regression and linear discriminant analysis (LDA) also demonstrated high performance (AUC = 0.994 for both), with κ values of 0.917 and 0.863, respectively.

**Table 5:** Machine learning performance metrics for predicting adiposity-defined obesity (10-fold stratified cross-validation).

Algorithm	AUC	AP	F1	Precision	Recall	Specificity	Brier	Kappa
MLP	0.999	0.999	0.983	0.985	0.981	0.987	0.013	0.967
Logistic Regression	0.994	0.994	0.956	0.962	0.954	0.964	0.032	0.917
LDA	0.994	0.994	0.923	0.988	0.864	0.993	0.055	0.863
Gradient Boosting	0.993	0.992	0.955	0.960	0.953	0.962	0.035	0.914
Random Forest	0.988	0.987	0.940	0.944	0.933	0.953	0.052	0.887
KNN	0.985	0.981	0.930	0.926	0.934	0.941	0.050	0.867
AdaBoost	0.981	0.979	0.918	0.919	0.916	0.933	0.197	0.845
Decision Tree	0.962	0.952	0.893	0.893	0.905	0.890	0.071	0.794
Extra Trees	0.945	0.942	0.848	0.908	0.798	0.899	0.120	0.721
Naive Bayes	0.850	0.851	0.724	0.740	0.709	0.762	0.167	0.504

AUC, area under the ROC curve; AP, average precision; Brier, Brier score; κ, Cohen’s kappa.

Across models, discrimination performance was consistently high. All algorithms achieved an AUC above 0.85, and seven of ten models exceeded an AUC of 0.98. Performance differences between top-performing models were relatively small, particularly among MLP, logistic regression, LDA, and gradient boosting.

The comparable performance observed between linear (logistic regression, LDA) and non-linear models suggests that a substantial proportion of the relationship between anthropometric variables and adiposity-defined obesity can be captured using relatively simple model structures.

Model performance was consistent across stratified analyses by sex and country, with high discrimination observed in all subgroups.

**Figure 7:**
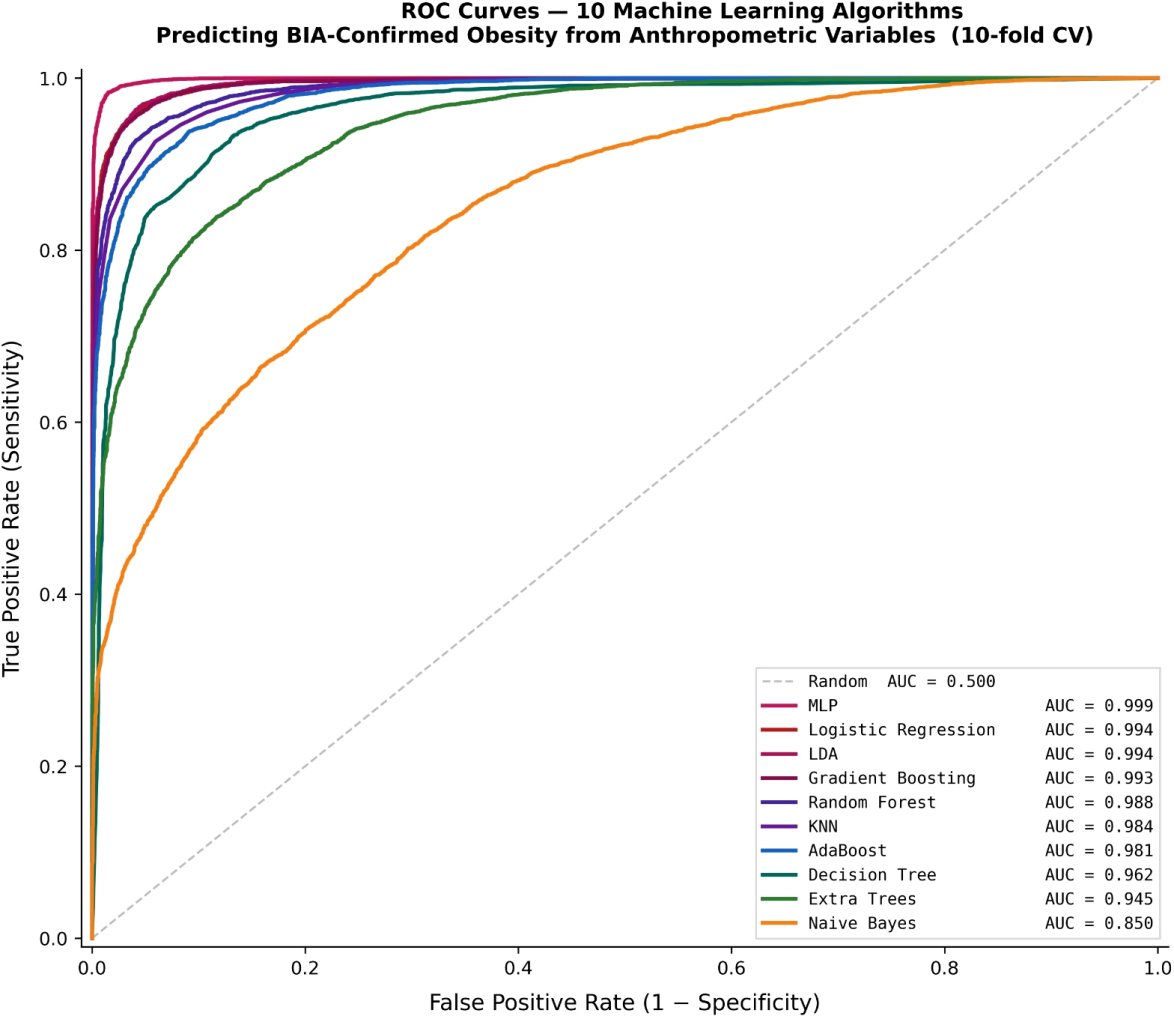
Receiver operating characteristic (ROC) curves for machine learning models predicting adiposity-defined obesity. All models show high discrimination performance relative to the random baseline. Top-performing models (MLP, logistic regression, LDA, and gradient boosting) demonstrate closely overlapping ROC curves.

**Figure 8:**
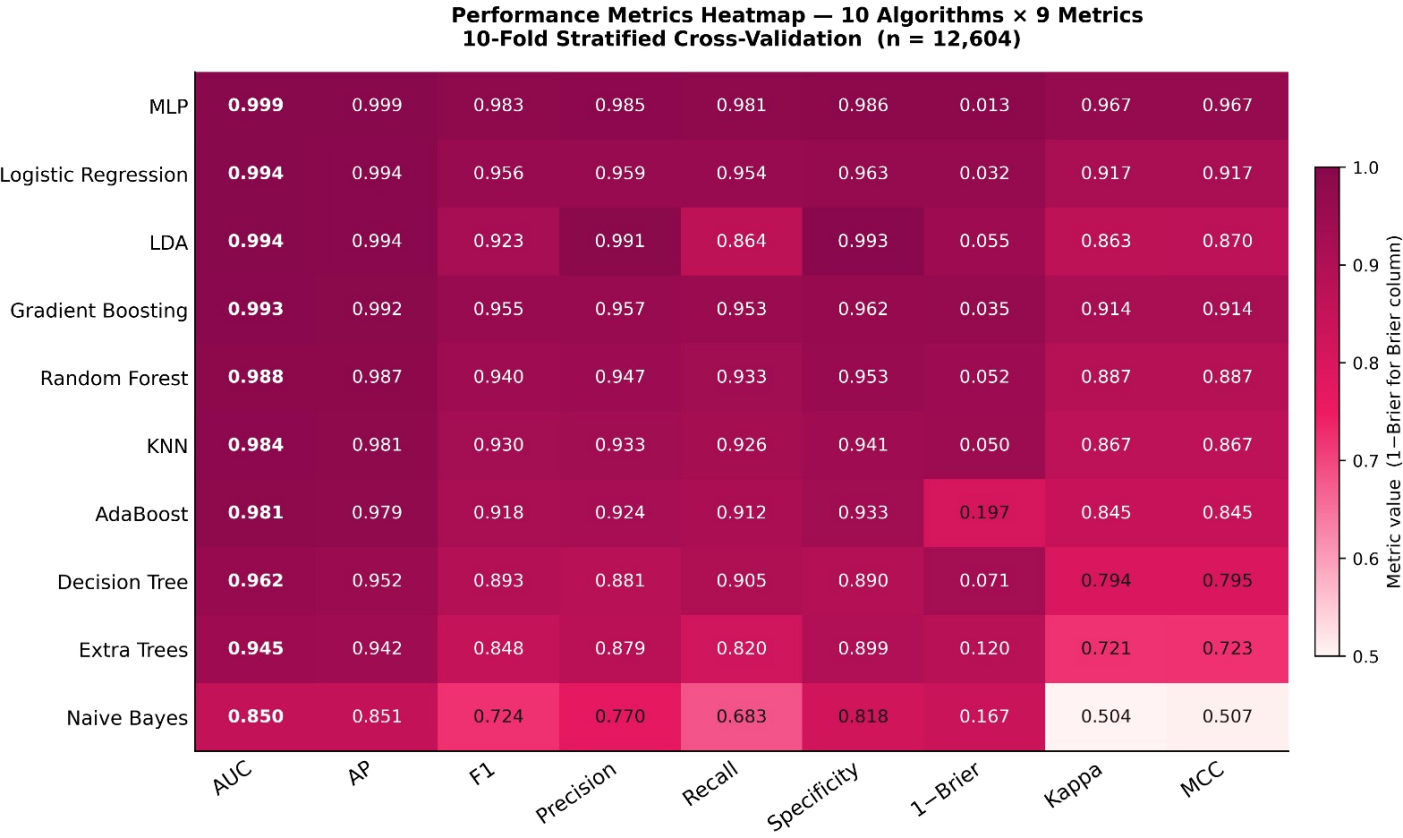
Heatmap of performance metrics across machine learning models. Color intensity reflects metric magnitude across models and evaluation criteria. Top-performing models show consistently high discrimination and agreement metrics.

**Figure 7** and **8** illustrate model discrimination and overall performance across algorithms. Consistent performance was observed in stratified analyses by sex and country, with high discrimination metrics maintained across subgroups.

Sensitivity analyses confirmed that the discriminatory performance of the models was not solely driven by the inclusion of BMI as a predictor. When BMI was removed and the predictor set restricted to age, body weight, height, basal metabolic rate, sex, and country, discrimination remained essentially unchanged (logistic regression AUC = 0.994 both with and without BMI; gradient boosting AUC ≈ 0.99 in both cases, consistent with the full-model values reported in **Table 5**). When both BMI and BMR were removed, leaving only age, body weight, height, sex, and country, discrimination decreased but remained substantial (logistic regression AUC = 0.908; gradient boosting AUC = 0.915). These results indicate that the high overall performance is not attributable to the mathematical dependency between BMI and its constituent variables alone, since omitting BMI did not materially degrade model performance, and that simple anthropometric variables retain meaningful predictive signal for adiposity-defined obesity even in the absence of a BMI input.

### 3.9 Feature importance analysis

**Figure 9:**
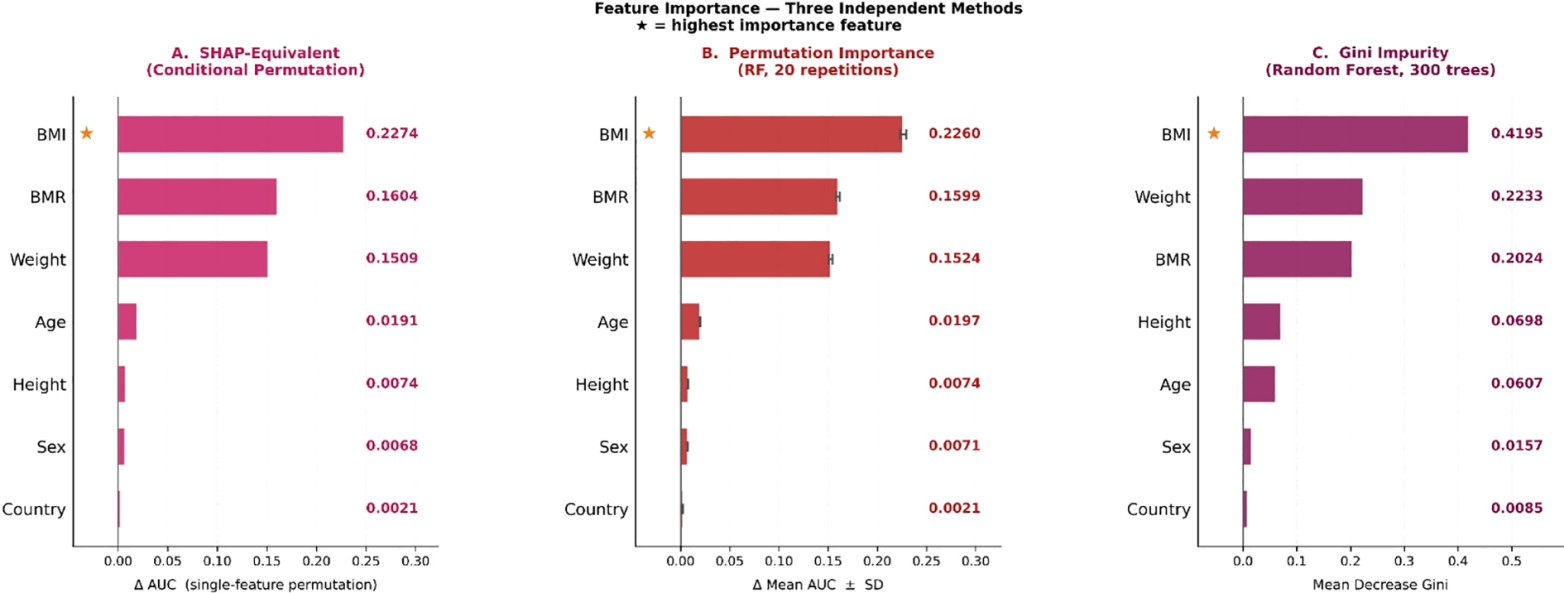
Feature importance derived from three complementary methods. **(A)** Conditional permutation-based feature importance estimated as ΔAUC after random permutation of each feature. **(B)** Permutation importance with mean and standard deviation across repeated iterations. **(C)** Gini impurity-based importance from a random forest model. A consistent ranking of predictors is observed across methods, with BMI, basal metabolic rate, and body weight contributing most to model performance.

Variable importance derived from three independent methods is presented in **Figure 9**. Across all approaches, BMI was consistently the most influential predictor.

BMI emerged as the most influential variable across methods, followed by basal metabolic rate and body weight, whose relative order varied across methods. Additional variables, including sex, height, and age, showed comparatively smaller contributions, while country had minimal influence on model performance.

The similarity in variable ranking across conditional permutation-based feature importance, permutation importance, and Gini impurity suggests a stable pattern of predictor contribution within the dataset.

### 3.10 Partial dependence and classification performance

**Figure 10:**
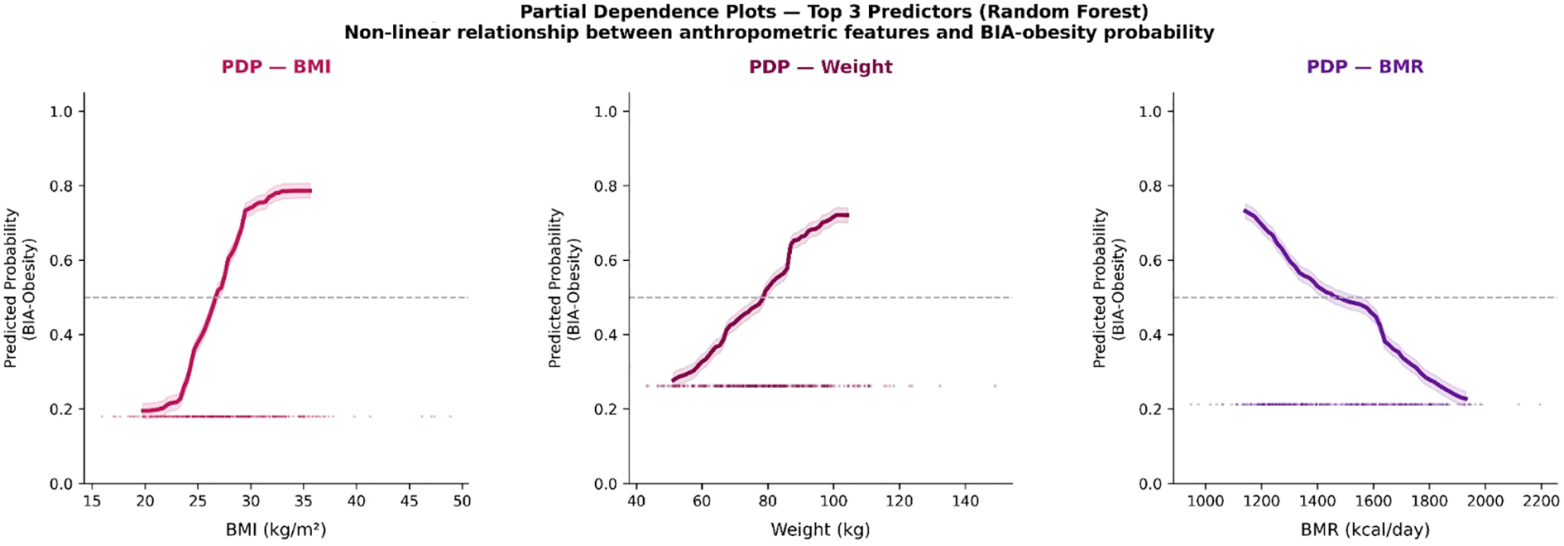
Partial dependence plots for the three most influential predictors. Plots show the marginal effect of BMI, weight, and BMR on predicted probability of adiposity-defined obesity using a random forest model. Non-linear relationships are observed, particularly for BMI and weight, while BMR shows an inverse association with predicted probability.

Partial dependence plots for the three most influential predictors are shown in **Figure 10**. For BMI, predicted probability of adiposity-defined obesity increased non-linearly, with a steeper transition observed between approximately 24 and 30 kg/m². A similar monotonic increase was observed for weight.

In contrast, basal metabolic rate (BMR) showed an inverse relationship, with lower values associated with higher predicted probability of adiposity-defined obesity. These patterns indicate that the relationship between anthropometric variables and predicted obesity probability is not strictly linear.

**Figure 11:**
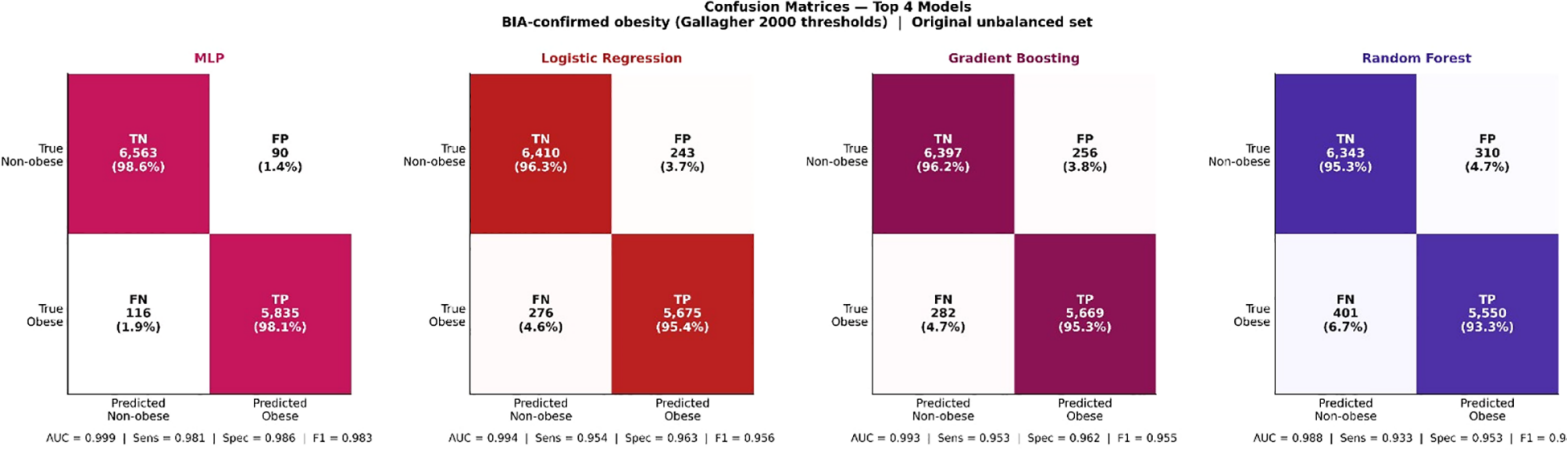
Confusion matrices for the four highest-performing models. Matrices display classification outcomes for adiposity-defined obesity. High overall accuracy is observed across models, with misclassifications concentrated near decision boundaries.

To further evaluate classification performance, confusion matrices for the four highest-performing models are presented in **Figure 11**. The multilayer perceptron (MLP) showed high classification accuracy, with similar patterns observed across other top-performing models.

Misclassifications were primarily observed among individuals located near classification boundaries, where BMI and BF% values show greater overlap. These regions correspond to areas of increased uncertainty in classification based on anthropometric variables alone.

**Figure 12:**
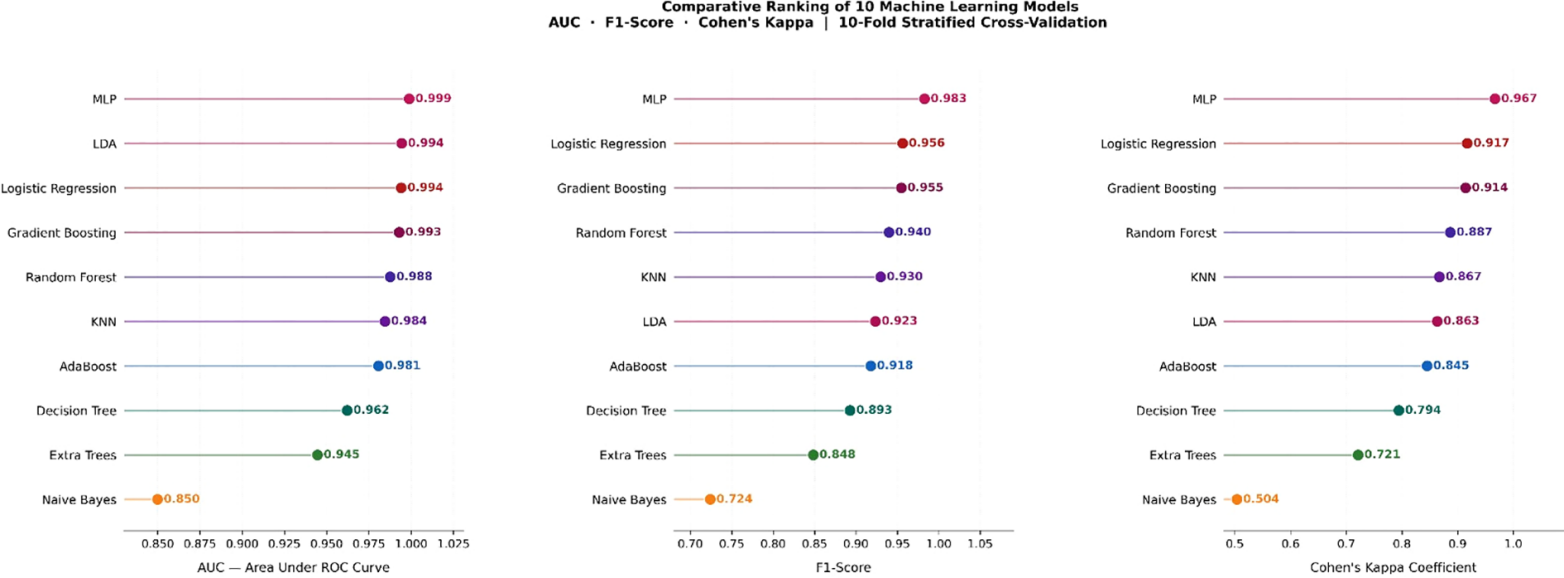
Comparative performance of machine learning models across multiple metrics. Models are ranked according to AUC, F1-score, and Cohen’s kappa. Top-performing models show consistently high performance across evaluation metrics, with relatively small differences between them.

Finally, a comparative ranking of model performance across multiple metrics is shown in **Figure 12**. Top-performing models, including MLP, logistic regression, and LDA, showed consistently high performance across AUC, F1-score, and Cohen’s kappa. Performance differences between these models were relatively small.

## 4 Discussion

The present study shows that BMI, when used as a stand-alone obesity screening tool, substantially underestimates excess adiposity in Latin American adults and fails to capture clinically relevant body composition phenotypes. In this large pooled cohort of 12,604 adults from Chile and Mexico, adiposity-defined obesity based on age- and sex-specific Gallagher thresholds was markedly more prevalent than BMI-defined obesity, generating a 23.7 percentage-point difference between classification systems. Agreement between BMI-defined and adiposity-defined obesity was only moderate, and BMI showed low sensitivity despite high specificity. In practical terms, BMI performed reasonably well when it identified an individual as obese, but it missed a substantial proportion of those with excess body fat according to BF%. This pattern is highly consistent with previous work showing that BMI is a pragmatic index of mass-for-height rather than a direct measure of adiposity. Romero-Corral et al. demonstrated in a large adult population that BMI ≥30 kg/m² had high specificity but limited sensitivity for diagnosing obesity defined by body fat percentage, and Gómez-Ambrosi et al. similarly showed that BMI classification misses a meaningful proportion of subjects with elevated adiposity and adverse cardiometabolic risk profiles (3, 24). Our findings extend this problem to a large Latin American cohort and indicate that the diagnostic limitations of BMI are not marginal, but structural.

This issue is particularly relevant in Latin America, where obesity and diet-related non-communicable disease have increased markedly over recent decades. Regional evidence has described rapid nutrition and epidemiologic transition characterized by increased intake of ultra-processed and energy-dense foods, declining physical activity, urbanization, and aging, all of which contribute to a rising adiposity burden (12, 13). In such a context, dependence on BMI alone is likely to underestimate the true prevalence of excess adiposity, especially when the objective is early detection of endocrine and cardiometabolic risk rather than simple categorization by weight-for-height. The present results support that concern directly. The gap between BMI-defined and adiposity-defined obesity was large, and this gap was not randomly distributed across the cohort. Rather, it was concentrated in specific discordant phenotypes and differed substantially by sex, suggesting that BMI-based surveillance may obscure a clinically important fraction of adults with excess body fat in Latin American settings.

A major clinical implication of our study is the frequency of normal-weight obesity. Among participants with BMI in the normal range, NWO was identified in 16.2% of women and 4.6% of men, with a strong female predominance across age groups. This phenotype is clinically meaningful because normal-weight obesity has repeatedly been associated with insulin resistance, dyslipidemia, low-grade inflammation, and increased cardiovascular risk despite the absence of overweight or obesity by BMI. De Lorenzo et al. first described NWO women as a subgroup with adverse cardiovascular risk indices and altered body composition despite normal body weight, and subsequent work showed early inflammatory alterations and cardiometabolic dysregulation in this phenotype (9, 25). Romero-Corral et al. later reported that NWO was associated with cardiovascular mortality, particularly in women, further establishing that a normal BMI cannot be assumed to reflect metabolic safety (10). More recent studies have reinforced the association between NWO and increased cardiometabolic risk in young adults and community cohorts (26). Our results are fully consistent with that literature and indicate that reliance on BMI alone is especially likely to miss women with clinically relevant excess adiposity.

The pronounced female predominance of NWO in this cohort is biologically plausible. Women typically have a higher body fat percentage than men at the same BMI and exhibit marked sex differences in adipose tissue quantity, distribution, and storage pattern. Karastergiou et al. emphasized that women have proportionally greater total adiposity and preferential subcutaneous and gluteofemoral fat deposition, whereas men tend to accumulate more visceral fat at lower total BF% (27). This sex dimorphism helps explain why the same BMI value may correspond to substantially different adiposity levels in women and men. Our findings therefore support the interpretation that BMI-based thresholds are particularly insensitive for detecting excess adiposity in women. At the same time, because hormonal variables were not assessed, these data should not be overinterpreted as evidence of menopause-related or endocrine-transition mechanisms; the robust conclusion is simply that sex-specific differences in body composition materially affect BMI classification performance.

Another important contribution of this study is the demonstration that a strong population-level association between BMI and BF% does not guarantee reliable individual-level classification. BMI and BF% were strongly correlated in both sexes, and OLS models explained a substantial proportion of BF% variance. However, the totality of the analytical framework showed that this global correlation masks clinically relevant discordance. First, the violin and box plots showed substantial overlap in BF% across adjacent BMI categories, particularly among women, indicating that BMI categories contain wide within-category heterogeneity in adiposity. Second, the quadrant analyses identified distinct and non-trivial clusters of normal-weight/high-adiposity and high-BMI/normal-adiposity individuals rather than isolated outliers. Third, residual diagnostics showed that the BMI–BF% relationship was not fully captured by a simple linear model, especially at the extremes of the distribution. Finally, Bland–Altman analysis demonstrated wide limits of agreement and patterns consistent with proportional bias, indicating that the discrepancy between BMI and BF% varies across the adiposity spectrum. Taken together, these findings show that BMI is informative at the population level but insufficiently precise as a stand-alone marker of adiposity at the individual level. This interpretation aligns with prior literature emphasizing the cardiovascular and metabolic heterogeneity of obesity and the substantial variation in body composition among individuals with the same BMI (8).

The OLS, residual, and Bland–Altman results are particularly important because they show that BMI misclassification is not simply random measurement noise. Residual and scale-location plots indicated non-uniform variance across fitted values, while Q–Q plots and Shapiro–Wilk tests showed clear departures from normality. Similarly, Bland and Altman’s framework reminds us that correlation does not imply interchangeability between two methods of measurement, and our wide limits of agreement indicate substantial individual-level disagreement even when average association is strong (28). The implication is that BMI compresses a multidimensional body composition space into a single scalar value, which creates structured error around clinically meaningful decision boundaries. This is especially problematic in endocrine and obesity research, where risk is increasingly recognized as depending not only on total body mass but also on fat quantity, regional distribution, and the balance between fat and lean tissue.

The high-BMI/normal-adiposity phenotype identified in our study requires careful interpretation. Earlier drafts referred to this subgroup as “metabolically healthy obese,” but that label is not appropriate here because metabolic biomarkers were not measured. A more accurate term is high-BMI/normal-adiposity phenotype. This distinction is methodologically important. The literature on high-BMI/normal-adiposity phenotype (metabolically healthy obesity) has shown that the concept is highly dependent on the definition used and that even individuals classified as metabolically healthy by conventional criteria may still have increased risk of cardiovascular disease and type 2 diabetes over time. In the Whitehall II cohort, Hinnouho et al. reported that high-BMI/normal-adiposity phenotype was associated with elevated cardiometabolic risk compared with metabolically healthy normal weight, and systematic reviews and meta-analyses have reached similar conclusions (29, 30). Accordingly, our data support the existence of a body composition phenotype characterized by elevated BMI without obesity-range BF%, particularly in men, but they do not support conclusions regarding long-term metabolic benignity.

The machine-learning analyses add a further conceptual layer to the study. The high discriminatory performance observed across all models indicates that the information needed to approximate adiposity-defined obesity is already embedded in simple anthropometric and related variables. In other words, the diagnostic failure of BMI does not appear to arise from a complete absence of measurable signal, but rather from the oversimplification of complex body composition into a single thresholded index. Importantly, the similarity in performance between more complex models and simpler linear methods such as logistic regression and LDA suggests that much of this information can be recovered using transparent and clinically implementable models. This is encouraging from a translational perspective, because it implies that meaningful improvement over BMI alone may be achievable without requiring black-box architectures. At the same time, the extremely high internal discrimination observed here must be interpreted cautiously. Internal cross-validation does not substitute for external validation, and inclusion of mathematically related predictors such as BMI, weight, and height may contribute to optimistic performance estimates if not carefully tested in sensitivity analyses.

The feature-importance analyses and partial dependence plots clarify why predictive performance was so strong. Across three complementary approaches, BMI emerged as the most influential predictor, followed by basal metabolic rate and body weight (their relative order varying across methods). This hierarchy is physiologically plausible. BMI remains strongly linked to total body size and adiposity, while body weight provides additional absolute mass information. The contribution of BMR is also coherent with known physiology, because resting or basal metabolic rate is closely related to fat-free mass and metabolically active tissue. Luke and Schoeller (31) demonstrated a close relationship between basal metabolic rate and fat-free mass during energy restriction, and De Lorenzo et al. (9) reported altered resting metabolic characteristics in women with normal-weight obesity. In that context, lower BMR values for a given anthropometric profile may indirectly reflect a lower proportion of metabolically active lean tissue relative to body size, thereby helping to distinguish individuals with higher fat burden that BMI alone does not identify. Our data do not demonstrate causality, but they do support the idea that BMR-related information can add clinically relevant signal to adiposity prediction models.

Viewed together, the phenotype prevalence data, BF% overlap across BMI strata, quadrant clustering, regression misfit, agreement analyses, and machine-learning results point in the same direction. BMI remains useful as a low-cost, universal first-pass anthropometric tool, but it should not be treated as a sufficient proxy for adiposity in adults. In this Latin American sample, BMI missed a large fraction of individuals with excess body fat, particularly women, and failed to represent important heterogeneity across the body composition spectrum. The present findings therefore support a more nuanced screening strategy in obesity and endocrinology research: one that preserves the practicality of conventional anthropometry but incorporates direct or model-based estimation of adiposity whenever possible.

## 5 Limitations

This study has several limitations that should be acknowledged. First, its cross-sectional design prevents causal inference and does not allow assessment of prospective metabolic, endocrine, or cardiovascular outcomes. Second, although the sample was large and included adults from two Latin American countries, it was not designed as a regionally representative epidemiologic survey; therefore, generalization to all Latin American populations should be made cautiously. Third, adiposity was assessed using multifrequency segmental BIA rather than criterion methods such as dual-energy X-ray absorptiometry or four-compartment models. Although BIA is widely used and considerably more informative than BMI for routine body composition assessment, its accuracy depends on standardized pre-measurement conditions, device-specific algorithms, and hydration status, and it may be less accurate at the extremes of body composition (5, 6, 18). Fourth, the Gallagher BF% thresholds, while widely cited and methodologically important, were not specifically developed in Latin American populations, and local recalibration against outcome-based or reference-method standards may further improve classification. Fifth, the absence of biochemical and hormonal biomarkers prevented direct metabolic phenotyping and precluded formal characterization of metabolically healthy or unhealthy obesity states. Finally, the machine-learning analyses were internally validated only. Because BMI, weight, and height are mathematically related, predictor redundancy may have contributed to the very high discrimination observed, and external validation in independent cohorts is essential before clinical implementation.

Two methodological caveats deserve specific elaboration. First, the choice of multifrequency segmental BIA as the reference for adiposity classification has implications that go beyond a generic accuracy disclaimer. Dual-energy X-ray absorptiometry (DXA) is generally regarded as the practical reference standard for body composition, and prior comparative work has shown that BIA and DXA can diverge systematically: BIA tends to underestimate fat mass in individuals with high adiposity and to overestimate it in lean individuals, with differences that depend on sex, hydration status, and the prediction equations embedded in each device. In the present cohort this means that the prevalence of adiposity-defined obesity and the magnitude of the BMI–BIA discordance should be interpreted as conditional on the InBody implementation of the Gallagher framework, and absolute prevalence estimates may shift modestly if DXA-based BF% thresholds were applied instead. The structural finding (that BMI systematically underdetects excess adiposity, especially in women) is unlikely to reverse under a DXA reference, since the BMI–BF% discordance pattern documented here is consistent with the DXA-based literature, but the precise magnitude of misclassification should not be over-interpreted as device-independent. Comparative calibration against DXA in a sub-sample is a clear priority for future work.

Second, regarding machine-learning validation, the AUC values approaching unity warrant explicit scrutiny for information leakage rather than reassurance from cross-validation alone. The outcome variable is derived from BF%, which is in turn estimated by the InBody algorithm from weight, height, age, and sex; these same variables appear as predictors. The sensitivity analyses presented in Section 3.8 partly mitigate this concern by showing that performance is preserved when BMI is removed and remains high (AUC ≈ 0.91) when BMR is also removed, indicating that the discriminatory signal is not solely an arithmetic artefact of the BMI definition. Nevertheless, internal cross-validation cannot rule out residual leakage arising from the device’s internal prediction equations, and the models reported here should be regarded as proof-of-concept rather than as deployment-ready screening tools. External validation in independent Latin American cohorts using DXA- or four-compartment-derived adiposity outcomes is required before any clinical implementation, and the present results are best framed as evidence that simple anthropometric variables retain substantial predictive signal for BIA-defined obesity, not as a claim of near-perfect generalizable accuracy.

A third caveat concerns dataset harmonization. The pooled cohort combines three datasets collected across two countries and two InBody models (370 and 720). Although both devices share the same eight-point tetrapolar electrode configuration, multifrequency segmental measurement principle, and standardized pre-test protocol, the InBody 370 and 720 use different proprietary frequency sets and prediction equations and have been validated against different reference methods in different populations. Because the two devices were used in geographically distinct sub-cohorts (InBody 370 in Chile, InBody 720 in Mexico), residual device-related variability cannot be statistically separated from country-related variability, and we cannot exclude that part of the country-level differences in BIA-defined obesity prevalence reflects between-device differences in BF% estimation rather than population biology alone. We retained country as a covariate in stratified analyses and as an input to the predictive models to partially absorb this joint effect, but a formal cross-calibration study of the two InBody models in the same individuals is needed to fully resolve this question. Consistent with this concern, BMI–BIA agreement diverged between sub-cohorts (Chile κ = 0.464, moderate; Mexico κ = 0.374, fair), and because device and country are completely confounded in the present design, this divergence should not be interpreted as a genuine population difference in the diagnostic behavior of BMI until the two InBody models are cross-calibrated in the same individuals.

## 6 Conclusions

The present study shows that BMI systematically underdetects excess adiposity and misclassifies relevant body composition phenotypes in Latin American adults when compared with adiposity-defined criteria derived from BIA. Agreement between BMI-defined and adiposity-defined obesity was only moderate, and BMI showed limited sensitivity despite high specificity. Misclassification was especially pronounced in women and was strongly reflected in the prevalence of normal-weight obesity, the overlap of BF% across BMI categories, and the structured discordance observed in regression and agreement analyses. These findings indicate that BMI remains useful for broad anthropometric screening but is insufficient as a stand-alone classifier of excess adiposity in this population. The study also shows that the diagnostic signal lost by BMI alone can be at least partially recovered using simple multivariable models based on routine anthropometric and metabolic variables.

## 7 Practical implications

From a practical standpoint, the results suggest that normal BMI should not be used as sufficient evidence to exclude excess adiposity, particularly in women. In clinical or research settings where body composition assessment is feasible, the addition of standardized BIA can substantially improve phenotypic classification beyond BMI alone. In resource-limited contexts where direct body composition assessment is unavailable, simple multivariable prediction tools based on age, sex, weight, height, BMI, and possibly BMR may offer a pragmatic intermediate solution. Because logistic regression and LDA performed similarly to more complex algorithms, future screening tools do not necessarily need to rely on black-box architectures to improve upon BMI-based classification. In endocrinology and obesity-focused practice, these findings support a shift toward screening strategies that better capture adiposity itself rather than body mass alone.

## 8 Future research directions

Future studies should externally validate these findings in independent and more representative Latin American cohorts, including diverse age ranges and ethnic subgroups. Comparative calibration against DXA and multi-compartment reference models would strengthen the body composition framework and help refine locally relevant BF% thresholds. Longitudinal studies are also needed to determine whether the discordant phenotypes identified here, especially NWO and high-BMI/normal-adiposity, differ in endocrine, metabolic, and cardiovascular outcomes over time. The addition of biochemical, inflammatory, and hormonal biomarkers would allow more rigorous characterization of metabolic health and improve interpretation of discordant phenotypes. Finally, implementation studies should assess whether transparent multivariable screening tools can improve clinical decision-making, risk stratification, and cost-effectiveness in primary care and public health settings compared with BMI alone.

## Data Availability

The raw data supporting the conclusions of this article will be made available by the authors, without undue reservation.
